# Identification of potential therapeutic targets for atherosclerosis by analysing the gene signature related to different immune cells and immune regulators in atheromatous plaques

**DOI:** 10.1186/s12920-021-00991-2

**Published:** 2021-06-03

**Authors:** Yang Shen, Li-rong Xu, Xiao Tang, Chang-po Lin, Dong Yan, Song Xue, Rui-zhe Qian, Da-qiao Guo

**Affiliations:** 1 Department of Vascular Surgery, Institute of Vascular Surgery, Zhongshan Hospital, Fudan University, 180 Fenglin Rd, Shanghai, 200032 China; 2 Department of Physiology and Pathophysiology, School of Basic Medical Sciences, Fudan University, 130 Dongan Rd, Shanghai, 200032 China

**Keywords:** Atherosclerosis, Immune infiltration, ESTIMATE algorithm, Circadian clock

## Abstract

**Background:**

Atherosclerosis is a chronic inflammatory disease that affects multiple arteries. Numerous studies have shown the inherent immune diversity in atheromatous plaques and suggest that the dysfunction of different immune cells plays an important role in atherosclerosis. However, few comprehensive bioinformatics analyses have investigated the potential coordinators that might orchestrate different immune cells to exacerbate atherosclerosis.

**Methods:**

Immune infiltration of 69 atheromatous plaques from different arterial beds in GSE100927 were explored by single-sample-gene-set enrichment analysis (presented as ssGSEA scores), ESTIMATE algorithm (presented as immune scores) and CIBERSORT algorithm (presented as relative fractions of 22 types of immune cells) to divide these plaques into ImmuneScoreL cluster (of low immune infiltration) and ImmuneScoreH cluster (of high immune infiltration). Subsequently, comprehensive bioinformatics analyses including differentially-expressed-genes (DEGs) analysis, protein–protein interaction networks analysis, hub genes analysis, Gene-Ontology-terms and KEGG pathway enrichment analysis, gene set enrichment analysis, analysis of expression profiles of immune-related genes, correlation analysis between DEGs and hub genes and immune cells were conducted. GSE28829 was analysed to cross-validate the results in GSE100927.

**Results:**

Immune-related pathways, including interferon-related pathways and PD-1 signalling, were highly enriched in the ImmuneScoreH cluster. HLA-related (except for HLA-DRB6) and immune checkpoint genes (IDO1, PDCD-1, CD274(PD-L1), CD47), RORC, IFNGR1, STAT1 and JAK2 were upregulated in the ImmuneScoreH cluster, whereas FTO, CRY1, RORB, and PER1 were downregulated. Atheromatous plaques in the ImmuneScoreH cluster had higher proportions of M0 macrophages and gamma delta T cells but lower proportions of plasma cells and monocytes (p < 0.05). CAPG, CECR1, IL18, IGSF6, FBP1, HLA-DPA1 and MMP7 were commonly related to these immune cells. In addition, the advanced-stage carotid plaques in GSE28829 exhibited higher immune infiltration than early-stage carotid plaques.

**Conclusions:**

Atheromatous plaques with higher immune scores were likely at a more clinically advanced stage. The progression of atherosclerosis might be related to CAPG, IGSF6, IL18, CECR1, FBP1, MMP7, FTO, CRY1, RORB, RORC, PER1, HLA-DPA1 and immune-related pathways (IFN-γ pathway and PD-1 signalling pathway). These genes and pathways might play important roles in regulating immune cells such as M0 macrophages, gamma delta T cells, plasma cells and monocytes and might serve as potential therapeutic targets for atherosclerosis.

**Supplementary Information:**

The online version contains supplementary material available at 10.1186/s12920-021-00991-2.

## Background

Increasing evidence has demonstrated that atherosclerosis (AS) is a systemic chronic inflammatory disease of the arterial wall that results from the accumulation of lipoprotein and the activation of diverse dysregulated immune cells [1–4]. Previous studies have also shown that the upregulation of the leukocyte levels of the N6-methyladenosine (m6A) modification and the disruption of circadian clocks are proatherogenic [5, 6]. In addition, the metabolic changes driven by rhythms of the circadian clock of immune cells could direct their immune output [7]. Although some immune cells have been proposed as potential therapeutic targets of atherosclerosis [8–10], the specific roles of different immune cells and the mechanism regulating their coordination with each other in atherosclerosis remain unclear. The molecular interactions between the circadian clocks and the immune system output in atherosclerosis are manifold and have not been fully documented. Furthermore, the role of the N6-methyladenosine (m6A) modification of different clock genes in different leukocytes in atherosclerotic lesions remains to be explored.

**Fig. 1:**
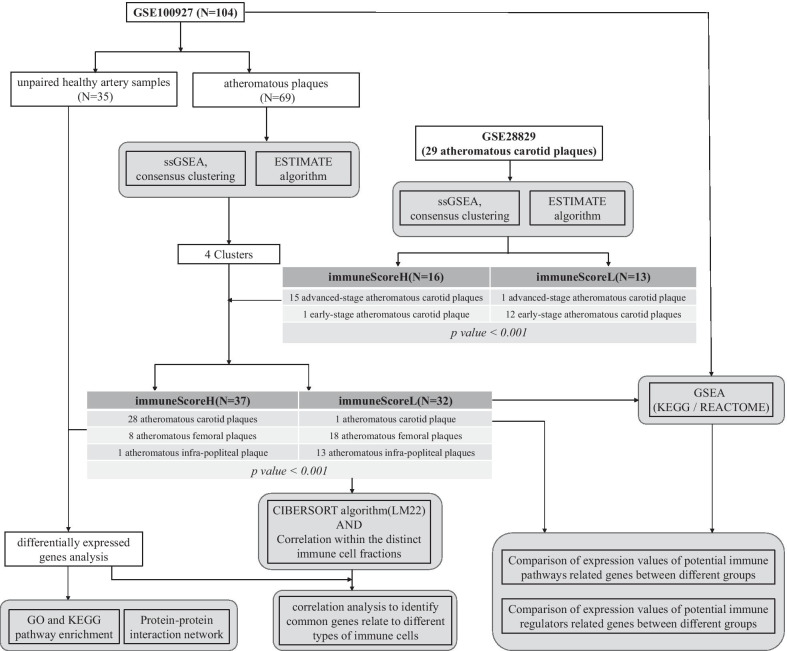
Analysis pipeline of expression values from microarrays. The expression values of GSE100927 and GSE28829 were analysed according to the pipeline. The atheromatous plaques in GSE100927 and GSE28829 were divided into a cluster with low immune scores (ImmuneScoreL cluster) and a cluster with high immune scores (ImmuneScoreH cluster) according to the degree of immune infiltration. The distribution of clinically proven early-stage and advanced-stage carotid plaques in GSE28829 was statistically significant (*p* < 0.001), and the distribution of atheromatous plaques from different peripheral arteries in GSE100927 was statistically significant (*p* < 0.001)

Given all the above-mentioned findings and problems, we hypothesized that more advanced stages of AS might be related to higher immune infiltration and different expression levels of immune checkpoint genes, m6A-related genes and circadian clock genes. Although the development of atherosclerosis in distinct vascular regions responds differently to common risk factors [11, 12] and the immune-related gene signatures show heterogeneity between different atherosclerotic lesions [13, 14], we presumed that some common genes or immune-related pathways might play important roles in the development of atherosclerosis. In this study, we conducted a comprehensive computational bioinformatic analysis using GSE100927 [14] and GSE28829 [15] to identify potential pathways and genes that might coordinate different immune cells to contribute to the progression of atherosclerosis (Fig. 1).

## Methods

### Data acquisition

The GSE100927 dataset was downloaded from the Gene Expression Omnibus (GEO) database (http://www.ncbi.nlm.nih.gov/geo/) [16]. The GSE100927 dataset includes 29 atheromatous carotid plaques, 26 atheromatous femoral plaques, 14 atheromatous infra-popliteal plaques, 12 control samples obtained from healthy carotid arteries, 12 control samples obtained from healthy femoral arteries, and 11 control samples obtained from healthy infra-popliteal arteries. Expression profiling arrays of GSE100927 were generated using GPL17077 (Agilent-039494 SurePrint G3 Human GE v2 8 × 60 K Microarray 039,381). Additionally, the GSE28829 dataset, which consists of 16 clinically proven advanced-stage atheromatous carotid plaques and 13 early-stage atheromatous carotid plaques, was downloaded from the GEO database. Expression profiling arrays of GSE28829 were generated using GPL570 (HG-U133_Plus_2, Affymetrix Human Genome U133 Plus 2.0 Array). The batch effects between different datasets or different groups or different samples were eliminated using the limma R package (version 3.40.6) [17].

### Estimation of the immune and stromal scores in atheromatous plaques

Single-sample gene-set enrichment analysis (ssGSEA) was conducted to analyse the immune cell types present in all the samples of the GSE100927 and GSE28829 datasets using the GSVA R package(version 1.32.0) [18]. The enrichment of an immune cell type meta-gene in a given sample was scored (ssGSEA score) based on a set of metagenes for 28 immune cell subpopulations [19]. Note that these enrichments should not be interpreted as deconvolutions of actual cell-type proportions. The presence of infiltrated immune cells and stromal cells in the atheromatous plaques was further evaluated by immune and stromal scores calculated using the ESTIMATE algorithm using gene-level expression data (Estimate R package, version 1.0.11) [20]. The different immune cell compositions in atheromatous plaques were assessed using the CIBERSORT algorithm and an LM22 leukocyte signature matrix as the input matrix of reference gene expression signatures [21]. The heterogeneity of immune infiltration among healthy artery samples and different atheromatous plaques and the correlation between different types of immune cells were explored.

### Consensus clustering and hierarchical cluster analysis of atheromatous plaques

The consensus clustering of different atheromatous plaques was performed using the ConsensusClusterPlus R package (version 1.48.0) based on the ssGSEA scores of infiltrated immune cells [22]. The Euclidean distances between samples were calculated. For the classification of atheromatous plaques into different subgroups, an unsupervised k-means clustering analysis using Euclidean distances was adopted for consensus clustering with 1000 repetitions. Hierarchical cluster analysis based on ssGSEA scores using Ward.D2 methods and Euclidean distances were performed for atheromatous plaques in each dataset (Stats R package, version 3.6.1).

### GO and KEGG pathway enrichment analysis

Fold changes (FCs) in gene expression values were calculated for atheromatous plaques in different groups, and the Benjamini–Hochberg method was used to adjust the original *p *values. The criteria |log2 FC|> 1 and adjusted *p* < 0.05 were used to identify the differentially expressed genes (DEGs) between the ImmuneScoreH cluster and other samples (including the healthy controls and ImmuneScoreL cluster) in GSE100927(limma R package, version 3.40.6). Gene Ontology (GO) and Kyoto Encyclopedia of Genes and Genomes (KEGG) pathway enrichment analyses of these DEGs were performed using the clusterProfiler R package(version 3.12.0) [23]. The GO annotation included biological process (BP), molecular function (MF), and cellular component (CC) categories. In addition, the DEGs between the ImmuneScoreH and ImmuneScoreL clusters in GSE100927 were identified using the same method.

### Construction of the protein–protein interaction (PPI) network

PPI networks of the DEGs between the ImmuneScoreH cluster and other samples (including the healthy controls and ImmuneScoreL cluster) in GSE100927 were constructed using the search tool for the retrieval of interacting genes (STRING database, V11.0; http://string-db.org/), which predicts protein functional associations and PPIs. After downloading the results from the STRING database with a confidence score > 0.9, Cytoscape software (V3.7.2; http://cytoscape.org/) was applied to visualize and analyse the biological networks and node degrees. Twelve algorithms on CytoHubba (version 0.1) were used to identify the hub genes. Genes with degree > 30 were identified as hub genes. These hub genes together with the DEGs between the ImmuneScoreH and ImmuneScoreL clusters in GSE100927 were analysed using Pearson correlation coefficients to identify common genes related to the relative percentages of immune cells in each sample.

### Gene set enrichment analysis (GSEA)

The expression profiles of healthy samples and atheromatous plaques and the expression profiles of different subtypes of atheromatous plaques in GSE100927 were used for GSEA using software provided by the Massachusetts Institute of Technology (version 4.1.0) [24]. The KEGG and REACTOME subsets of canonical pathways (CPs) of MSigDB (V7.1, https://www.gsea-msigdb.org/gsea/msigdb/index.jsp), which contains gene sets derived from the KEGG and Reactome pathway databases, were used as the a priori knowledge for the GSEA. The NES and a FDR < 0.25 were used to quantify the enrichment magnitude and statistical significance.

### Expression profiles of genes of interest

The expression profiles of HLA molecules, immune checkpoint molecules, m6A regulators, circadian rhythm-related genes, and IFN-γ signalling pathway-related genes (Additional file 6: Supplementary Table 1) were explored and compared between healthy artery samples and atheromatous plaques. The expression profiles of these genes were also explored and compared between different clusters of atheromatous plaques.

### Statistical analysis

All statistical analyses in this study were performed using R version 3.6.1. The expression profiles of genes of interest or predefined gene sets between clusters were compared using the Mann–Whitney-Wilcoxon test. The correlation among variables was evaluated with the Pearson correlation coefficient. Fisher’s exact test was used for nominal variables. A *p* value < 0.05 was considered statistically significant. Where appropriate, p values were corrected for multiple testing using the Benjamini–Hochberg false discovery rate method.

## Results

### Subtypes of atheromatous plaques in GSE100927

**Fig. 2:**
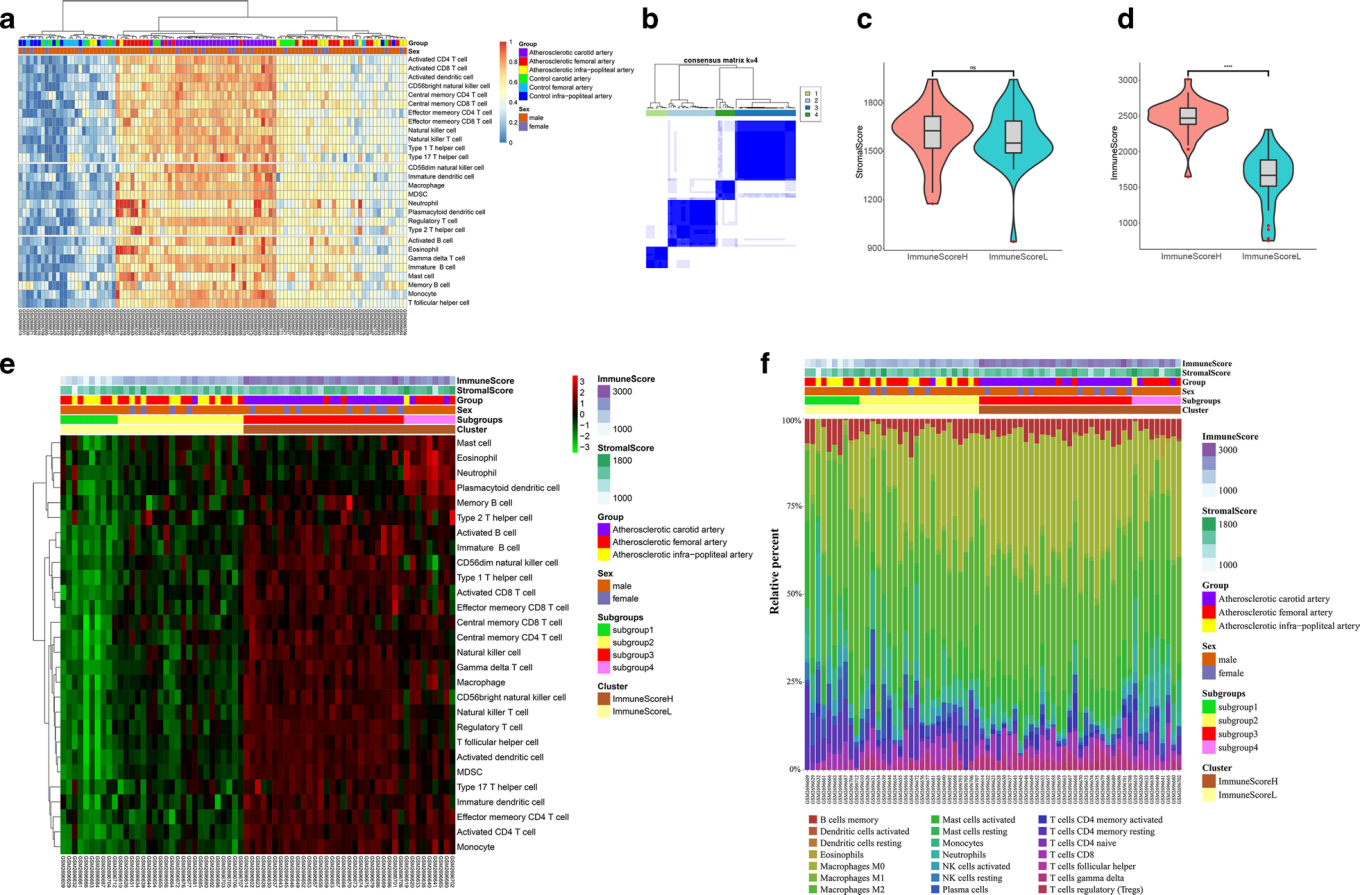
Atherosclerosis subtypes and distinct infiltrated immune cells in the samples in the GSE100927 dataset. **A** Heatmap of ssGSEA scores of all 104 samples in GSE100927 that were clustered based on the Euclidean distance using the Ward.D2 method. **B** Consensus clustering of ssGSEA scores of 69 atheromatous plaques based on the Euclidean distance using the k-means clustering method with k = 4. **C**, **D** Violin plots of stromal scores and immune scores in the plaques with high immune scores (ImmuneScoreH cluster) and the plaques with low immune scores (ImmuneScoreL cluster) of the 69 atheromatous plaques based on the ESTIMATE algorithm. **E** Heatmap of ssGSEA scores of four subgroups (subgroup1-4 which were divided using the k-means clustering method with k = 4 in **B**) and two clusters (ImmuneScoreH cluster and ImmuneScoreL cluster based on the immune score calculated by ESTIMATE algorithm) of the 69 atheromatous plaques in GSE100927. **F** Immune infiltration landscape in the four subgroups and two clusters of 69 atheromatous plaques demonstrated in **E**

The gene expression profiles of 69 atheromatous plaques and 35 healthy samples in the dataset GSE100927 were obtained from the GEO database. The relationship between these samples was evaluated by principal component analysis (PCA), and the intergroup distances were greater than the inner-group distances (Additional file 1: Figure S1A). The enrichment of an immune cell type meta-gene in a given sample was scored (ssGSEA score). Hierarchical clustering of the ssGSEA scores showed that all 104 samples were distributed into three distinct groups: a healthy group, a low immune infiltration group, and a high immune infiltration group. Healthy artery samples were mainly gathered into the healthy group, and atheromatous plaques were mainly gathered into the low and high immune infiltration groups. Notably, the atheromatous carotid plaques had higher ssGSEA scores than other plaques (Fig. 2A). An unsupervised consensus clustering analysis was performed to classify all 69 atheromatous plaques in GSE100927 based on their ssGSEA scores, and the cumulative distribution function (CDF) curve showed that k = 4 was an optimal choice (Additional file 1: Figure S1B–H). Therefore, the 69 atheromatous plaques in GSE100927 were grouped into four distinct subgroups (Fig. 2B). Based on the immune scores and stromal scores calculated using the ESTIMATE algorithm (Fig. 2C, D), the four subgroups (Fig. 2B, subgroup1/2/3/4 in Fig. 2E) of the 69 atheromatous plaques were divided into a cluster with high immune scores (ImmuneScoreH cluster) and a cluster with low immune scores (ImmuneScoreL cluster) (Fig. 2E). We found that all but one of the atheromatous carotid plaques were in the ImmuneScoreH cluster. Furthermore, we evaluated the fraction of immune cells in different groups of atheromatous plaques using the CIBERSORT algorithm. Figure 2F showed the different distributions of 21 types of immune cells in the 69 atheromatous plaques. The atheromatous plaques in the ImmuneScoreH cluster had a higher proportion of M0 macrophages and gamma delta T cells but a lower proportion of plasma cells and monocytes (Additional file 2: Figure S2A, B). Moreover, plasma cells were positively correlated with resting mast cells (r = 0.55, *p* < 0.001, Additional file 2: Figure S2C), and M0 macrophages were negatively correlated with activated NK cells (r = − 0.63, *p* < 0.001, Additional file 2: Figure S2C).

### Subtypes of atheromatous plaques in GSE28829

To explore whether results of unsupervised K-means clustering (using ssGSEA scores as input) is consistent with clustering results of immune scores (calculated by ESTIMATE algorithm) in a dataset with small sample size(n < 30), and to explore whether higher immune infiltration is correlated with more advanced atherosclerosis, we analysed the immune infiltration of atheromatous plaques in GSE28829. As shown in Additional file 3: Figure S3A, the 29 atheromatous plaques in GSE28829 were grouped into two distinct groups based on the ssGSEA analysis. The cluster dendrogram also showed that all the samples were divided into two groups (Additional file 3: Figure S3B). An unsupervised consensus clustering analysis of all 29 atheromatous plaques in GSE28829 based on the ssGSEA score showed that k = 2 was an optimal choice (Additional file 3: Figure S3C, D, subgroup1/2 in Additional file 3: Figure S3E). Based on the immune scores and stromal scores calculated using the ESTIMATE algorithm, the 29 atheromatous plaques were divided into the ImmuneScoreH and ImmuneScoreL clusters (Additional file 3: Figure S3E). The distribution of clinically proven early-stage and advanced-stage atheromatous plaques in the two clusters was statistically significant (Fig. 1). The ImmuneScoreL cluster of atheromatous plaques in GSE100927 (Additional file 2: Figure S2A) and GSE28829 (Additional file 3: Figure S3F) contained relatively higher proportions of plasma cells (*p* < 0.05) and monocytes (*p* < 0.05). In contrast, M0 macrophages (*p* < 0.05) and gamma delta T cells (*p* < 0.05) were present at relatively higher proportions in the ImmuneScoreH cluster. To further demonstrate the immune infiltration in health controls to atheromatous plaques derived from different datasets, we pooled GSE28829 and GSE100927. After removing batch effects, ssGSEA socres of different immune cell types in these pooled samples were calculated. Healthy arteries were of lower immune infiltration (Additional file 3: Figure S3G).

### Gene set enrichment analysis (GSEA) of GSE100927

Multiple immune-related pathways were highly enriched in atheromatous plaques (vs. healthy artery samples, Additional file 3: Fig. 3A–F), and these pathways included the B cell receptor signalling pathway (normalized enrichment score (NES) = 1.5637, false discovery rate (FDR) = 0.2085), leukocyte transendothelial migration (NES = 1.4851, FDR = 0.1689), natural killer cell-mediated cytotoxicity (NES = 1.5498, FDR = 0.1852), T cell receptor signalling pathway (NES = 1.5517, FDR = 0.1952), primary immunodeficiency (NES = 1.4222, FDR = 0.1732), and antigen processing and presentation (NES = 1.5726, FDR = 0.2379). These six immune-related pathways were also highly enriched in the ImmuneScoreH group (vs. the ImmuneScoreL group, Additional file 4: Fig. 4A–F). In addition, interferon-related pathways (Fig. 4G–J), PD-1 signalling (Fig. 4K, NES = 1.4343, FDR = 0.1613) and neutrophil degranulation (Fig. 4L, NES = 1.6372, FDR = 0.1153) were highly enriched in the ImmuneScoreH group (vs. the ImmuneScoreL group). To cross-validate these findings, we also performed GSEA for GSE28829. We found that these pathways were also highly enriched in the ImmuneScoreH cluster (vs. the ImmuneScoreL cluster) of GSE28829 (Additional file 4: Figure S4A–L).

**Fig. 3:**
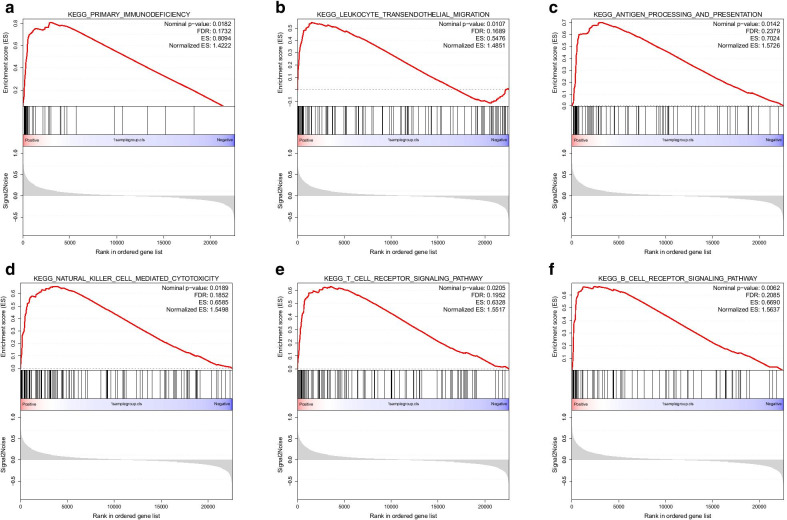
Gene set enrichment analysis (GSEA) comparing atheromatous plaques with healthy control samples of GSE100927. KEGG canonical pathways were used as the a priori information for the GSEA. **A** Primary immunodeficiency, **B** leukocyte transendothelial migration, **C** antigen processing and presentation, **D** natural killer cell-mediated cytotoxicity, **E** T cell receptor signalling pathway, and **F** B cell receptor signalling pathway were highly enriched in atheromatous plaques

**Fig. 4:**
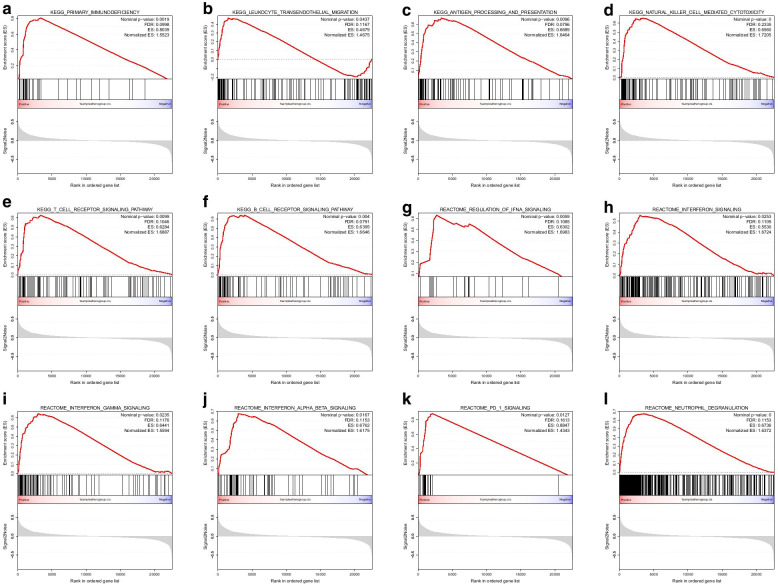
Gene set enrichment analysis (GSEA) comparing atheromatous plaques in the ImmuneScoreH cluster with those in the ImmuneScoreL cluster of GSE100927. **A**–**F** KEGG canonical pathways were used as the a priori knowledge for the GSEA. **A** Primary immunodeficiency, **B** leukocyte transendothelial migration, **C** antigen processing and presentation, **D** natural killer cell-mediated cytotoxicity, **E** T cell receptor signalling pathway, and **F** B cell receptor signalling pathway were highly enriched in the ImmuneScoreH cluster. **G**–**L** The REACTOME subset of canonical pathways was used as the a priori information for the GSEA. **G**–**J** Interferon-related pathways, **K** PD-1 signalling, and **L** neutrophil degranulation were highly enriched in the ImmuneScoreH cluster

### Correlation between atherosclerosis and genes of interest in GSE100927

**Fig. 5:**
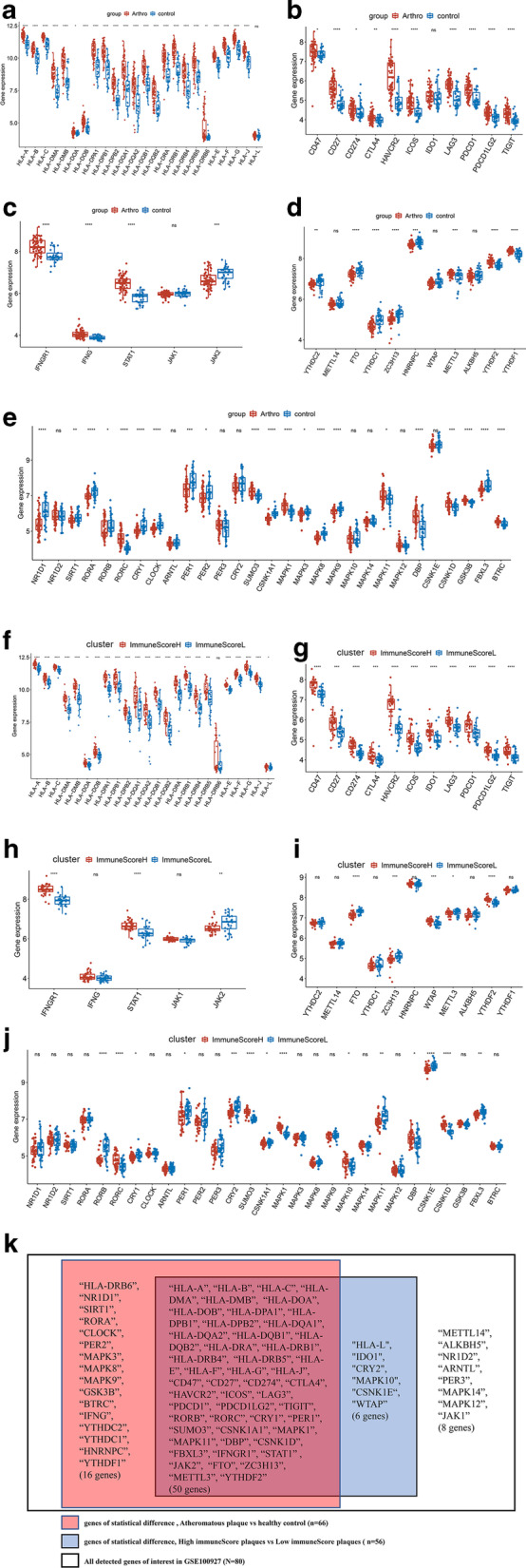
Comparison of the expression of genes related to different immune regulators in GSE100927. ns: *p* > 0.05, *p <  = 0.05, **p  ≤ 0.01, ***p  ≤ 0.001, ****p  ≤ 0.0001. 80 genes were detected in GSE100927. **A–E** were a set of comparisons between atheromatous plaques and healthy control: **A** box plot comparing the expression of human leukocyte antigen (HLA)-related genes, **B** immune checkpoint-related genes, **C** IFN-γ pathway marker genes, **D** m6A methylation regulator-related genes, **E** circadian rhythm-related genes. **F**–**J** were a set of comparisons between atheromatous plaques in the ImmuneScoreH cluster with that in atheromatous plaques in the ImmuneScoreL cluster: **F** Box plot comparing the expression of HLA-related genes, **G** immune checkpoint-related genes, **H** IFN-γ pathway marker genes, **I** m6A methylation regulator-related genes, **J** circadian rhythm-related genes. **K** Summarized the consistent results between the two sets of comparisons (atheromatous plaques vs. healthy controls/ImmunceScoreH cluster vs. ImmunceScoreL cluster)

Based on the above-presented results, we further explored immune-related genes, including HLA molecule-related genes, immune checkpoint-related genes, IFN-γ pathway-related genes, circadian rhythm-related genes, and m6A methylation regulator-related genes (Additional file 6: Supplementary Table 1). Eighty of these genes were detected in GSE100927 (Fig. 5). The expression of these 80 genes were compared between atheromatous plaques and healthy control artery samples (Fig. 5A–E), as well as between plaques of high immune scores (ImmuneScoreH cluster) and low immune scores (ImmuneScoreL cluster) (Fig. 5F–J). Fifty out of these 80 genes showed the same trends in both sets of comparisons and were statistically different, including HLA-DPA1, PDCD-1, CD274(PD-L1), CD47, IFNGR1, STAT1, JAK2, FTO, RORB, RORC, CRY1 and PER1. Only eight genes were of no statistical difference in both sets of comparisons (Fig. 5k). These findings were cross-validated in GSE28829. Thirty-four genes of interest were statistically different between immuneScoreH cluster and immuneScoreL cluster in GSE28829 (Additional file 5: Figure S5A–E). Thirty-one of these 34 genes showed the same trends when compared between immuneScoreH cluster and immuneScoreL cluster in GSE100927 (Additional file 5: Figure S5F).

### Identification of the DEGs and hub genes of GSE100927

**Fig. 6:**
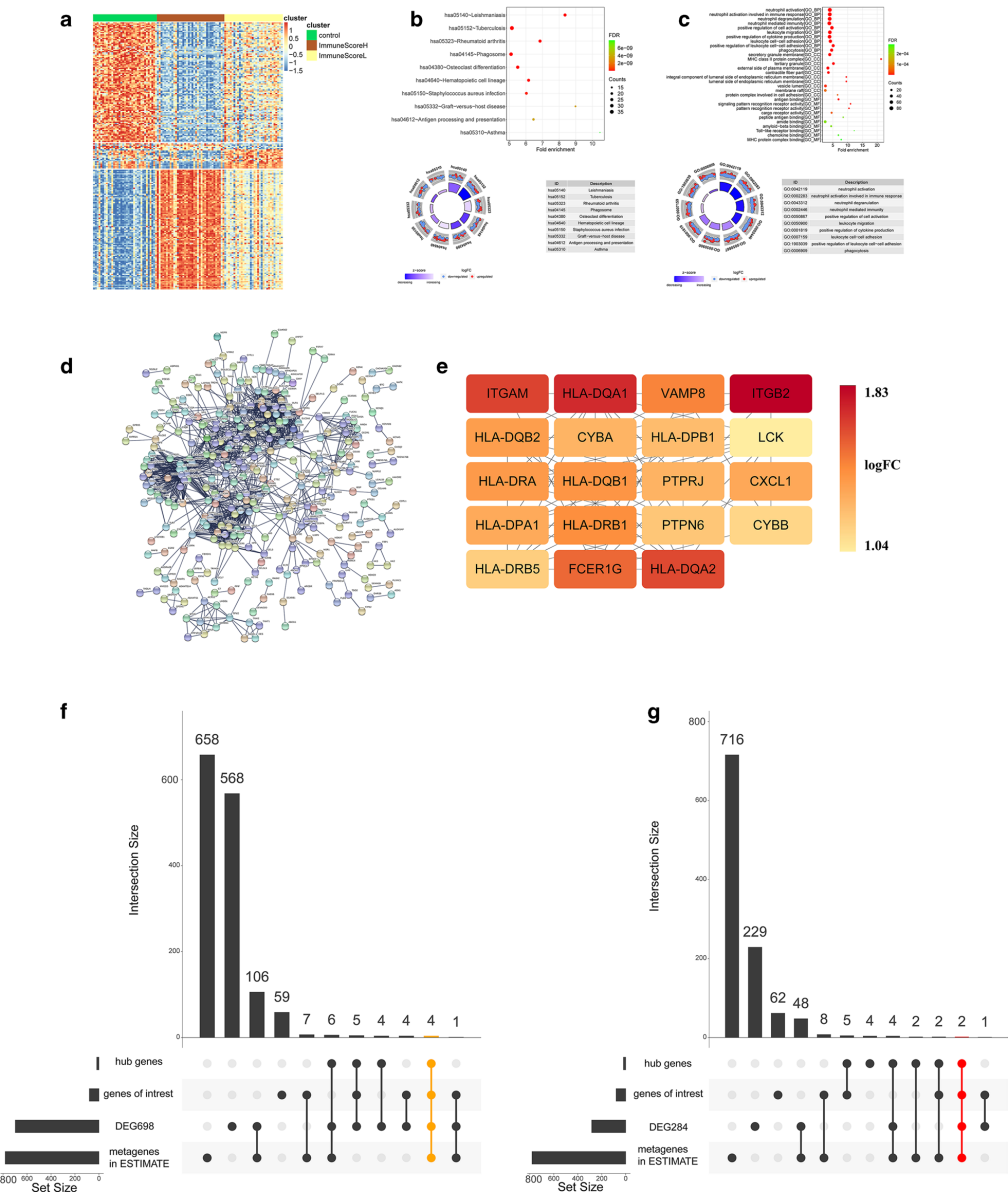
Differentially expressed genes (DEGs) and hub genes in the ImmuneScoreH cluster (vs. the ImmuneScoreL cluster and healthy controls). FDR: false discovery rate, logFC: log(fold change). **A** Heatmaps of DEGs among healthy control samples and atheromatous plaques in the ImmuneScoreL and ImmuneScoreH clusters. **B** Bubble and circle plots showing the results from the KEGG pathway enrichment analysis of DEGs of atheromatous plaques in the ImmuneScoreH cluster (vs. ImmuneScoreL cluster and healthy controls). The top 10 KEGG pathways ranked by the FDR are shown. **C** Bubble and circle plots showing the results from the Gene Ontology (GO) enrichment analysis, including the biological process (BP), molecular function (MF), and cellular component (CC) categories, of DEGs of atheromatous plaque in the ImmuneScoreH cluster (vs. ImmuneScoreL cluster and healthy controls). The top 10 BP, MF and CC terms ranked by the FDR are shown. **D** PPI network of DEGs of plaques in the ImmuneScoreH cluster. **E** Nineteen hub genes of plaques in the ImmuneScoreH cluster. **F** UpSet plot demonstrating overlaps between the 698 DEGs (ImmuneScoreH cluster *vs.* other samples, Additional file 7: Supplementary Table 2), the 19 hub genes (**E**), 80 detected genes of interest (Fig. 5**K**) and 782 metagenes in ESTIMATE. **G** UpSet plot demonstrating overlaps between the 284 DEGs (ImmuneScoreH cluster *vs.* ImmuneScoreL cluster, Additional file 8: Supplementary Table 3), 19 hub genes (**E**), 80 detected genes of interest (Fig. 5**K**) and 782 metagenes in ESTIMATE. HLA: human leukocyte antigen, PTPN6: protein tyrosine phosphatase non-receptor type 6, VAMP8: vesicle-associated membrane protein 8, ITGB2: integrin subunit beta 2, ITGAM: integrin subunit alpha M, CXCL1: C-X-C motif chemokine ligand 1, CYBA: cytochrome B-245 alpha chain, CYBB: cytochrome B-245 beta chain (also known as NADPH oxidase 2), FCER1G: Fc receptor gamma chain, LCK: lymphocyte cell-specific protein-tyrosine kinase, PTPRJ: protein tyrosine phosphatase receptor type J (also known as SCC1 or DEP1)

The comparison of the ImmuneScoreH cluster with the other samples (including the healthy controls and ImmuneScoreL cluster) identified 698 DEGs (Additional file 7: Supplementary Table 2, 216 upregulated DEGs and 482 downregulated DEGs) (Fig. 6A). Most of the DEGs in the ImmuneScoreH cluster were enriched in KEGG pathways such as haematopoietic cell lineage and phagosome (Fig. 6B). In addition, most of these DEGs were enriched in biological functions such as neutrophil activation, neutrophil degranulation, neutrophil-mediated immunity, and leukocyte migration (Fig. 6C). The protein–protein interaction network of these genes is shown in Fig. 6D. In addition, 19 hub genes (PTPN6, HLA-DRA, HLA-DRB1, VAMP8, ITGB2, ITGAM, CXCL1, CYBB, FCER1G, CYBA, HLA-DRB5, HLA-DQB1, HLA-DQA1, HLA-DQB2, HLA-DPB1, HLA-DPA1, LCK, HLA-DQA2, and PTPRJ; all with degrees > 30) were identified in the ImmuneScoreH cluster (Fig. 6E). The overlaps between the 698 DEGs, 19 hub genes, 80 detected genes of interest and 782 metagenes of ESTIMATE were shown in Fig. 6F. In addition, 284 DEGs (177 upregulated DEGs and 107 downregulated DEGs) were identified in the ImmuneScoreH cluster compared with the ImmuneScoreL cluster (Additional file 8: Supplementary Table 3). The overlaps between the 284 DEGs, 19 hub genes, 80 detected genes of interest and 782 metagenes in ESTIMATE were shown in Fig. 6G.

### Identification of common genes related to the relative percentages of immune cells in GSE100927

**Fig. 7:**
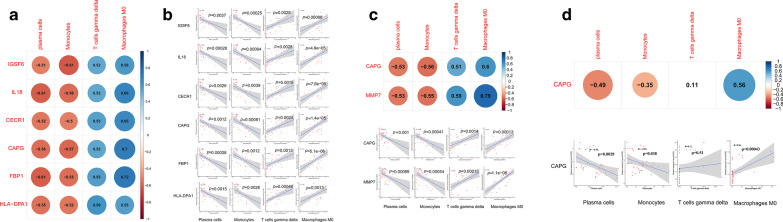
Identification of common genes related to plaques in the ImmuneScoreL and ImmuneScoreH clusters. CAPG: actin regulatory protein CAP-G, IGSF6: immunoglobulin superfamily member 6, IL18: interleukin-18, CECR1: cat eye syndrome critical region protein 1, FBP1: fructose-bisphosphatase 1, MMP7: matrix metallopeptidase 7. **A** Pearson correlation coefficients obtained for the correlations of CAPG, CECR1, IL18, IGSF6, and FBP1 with the relative proportions of M0 macrophages, gamma delta T cells, plasma cells and monocytes in the ImmuneScoreL cluster. **B** Scatterplot showing the correlations of the relative expression levels of CAPG, CECR1, IL18, IGSF6, and FBP1 to the relative proportions of M0 macrophages, gamma delta T cells, plasma cells and monocytes in the ImmuneScoreL cluster. **C** Pearson correlation coefficients obtained for the correlations of CAPG and MMP7 with the relative proportion of M0 macrophages, gamma delta T cells, plasma cells and monocytes in the ImmuneScoreH cluster. **D** Scatterplot showing the correlations of the relative expression levels of CAPG and MMP7 to the relative proportions of M0 macrophages, gamma delta T cells, plasma cells and monocytes in the ImmuneScoreH cluster. **E** Pearson correlation coefficients obtained for the correlations of CAPG with the relative proportion of M0 macrophages, gamma delta T cells, plasma cells and monocytes in the healthy controls. **F** Scatterplot showing the correlations of the relative expression levels of CAPG to the relative proportions of M0 macrophages, gamma delta T cells, plasma cells and monocytes in the healthy controls. The grey-shaded areas in the scatterplots represent the standard errors of the regression lines. R: correlation coefficient. The p values of all these genes were < 0.05 except for the correlation of the relative expression levels of CAPG to the relative proportions of gamma delta T cells in **F**

In the ImmuneScoreL cluster, actin regulatory protein CAP-G (CAPG), immunoglobulin superfamily member 6 (IGSF6), interleukin-18 (IL18), cat eye syndrome critical region protein 1 (CECR1), fructose-bisphosphatase 1 (FBP1), and HLA-DPA1 were positively related to the proportion of M0 macrophages and gamma delta T cells but negatively related to the proportion of plasma cells or monocytes (Fig. 7A, B). In the ImmuneScoreH cluster, CAPG and matrix metallopeptidase 7 (MMP7) were positively related to the proportion of M0 macrophages and gamma delta T cells but negatively related to the proportion of plasma cells or monocytes (Fig. 7C, D). The relationships were the same for the CAPG and plasma cells / monocytes / M0 macrophages in the health samples (Fig. 7E, F).

## Discussion

Atherosclerosis is a systemic chronic inflammatory disease associated with activated innate immune response [25]. A better understanding of immune infiltration in different plaques might provide a better understanding of atherosclerosis. In our study, we first deemed that the ESTIMATE algorithm might be appropriate for assessing the immune infiltration of different plaques because the gene sets used for estimating stromal and immune scores in the ESTIMATE algorithm are filtered by a dataset of gene expression in normal tissues (GSE1133, including smooth muscle, endothelium, heart, and haematopoietic cells) [20]. Then, an unsupervised consensus clustering of ssGSEA scores and the ESTIMATE algorithm were used successively to classify the plaques. As expected, in GSE100927, ImmuneScoreH cluster has higher ssGSEA scores than ImmuneScoreL group, indicating that the clustering of immune scores calculated by ESTIMATE algorithm were consistent with the unsupervised consensus clustering of ssGSEA score (Fig. 2E). Meanwhile, the results obtained from the application of the ESTIMATE algorithm to GSE28829, a dataset with small sample size (n = 29), were consistent not only with the unsupervised consensus clustering results but also with the different clinical stages of these carotid plaques (Additional file 3: Figure S3E), reinforcing the feasibility of applying ssGSEA and ESTIMATE algorithm to the classification of atheromatous plaques. In addition, the analysis of GSE100927 gathered most plaques from the infrapopliteal and femoral arteries into clusters different from those that included most plaques from the carotid artery (Fig. 2E), which indicated the distinct heterogeneity of immune infiltration in different atherosclerotic lesions. These findings suggest that plaques with higher immune infiltration might be at a more advanced stage of atherosclerosis.

In this study, we identified 12 immune-related pathways that might play important roles in atherosclerosis development by GSEA: seven pathways were related to immune cells, four pathways were related to interferon, and the other pathway was related to PD-1 signalling. These results, together with those from previous studies that explored the function of IFN-γ [26, 27] and PD-1/PD-L1 inhibitors [28], suggest that the inhibition of IFN-γ and PD-1/PD-L1 might reduce atherosclerosis. In addition, nine of the 19 hub genes belonged to major histocompatibility complex (MHC) class II (HLA-DP, HLA-DQ, and HLA-DR), which present antigens from outside a cell to T lymphocytes. All these results indicate that the IFN-γ pathway and PD-1 signalling pathway might serve as potential targets in atherosclerosis therapy.

Many types of immune cells have been proven to possess intrinsic clocks, and these cell types include macrophages [29], monocytes [30], neutrophils [31], natural killer (NK) cells [32], mast cells and eosinophils [33]. Circadian clock and m6A for the function of the circadian clock have emerged as important gatekeepers for different immune functions [34, 35]. In this study, we revealed that RORB, RORC, PER1, CRY1, FTO were in close relationship with atherosclerosis, particularly in atherosclerotic lesions with higher immune infiltration. While it’s been reported that the demethylase FTO (alpha-ketoglutarate-dependent dioxygenase) coimmunoprecipitates with CRY1/2 [36], studies also showed that upregulation of FTO and CRY1 attenuated atherosclerosis through macrophages and proinflammatory factors respectively [37, 38]. It was also reported that receptor tyrosine kinase-like orphan receptor (ROR) inverse agonist could induce an anti-atherogenic immune profile to decrease plaque formation [39]. Besides, loss of Per1 enhanced the recruitment of macrophages through an increase in CC chemokine receptor 2 (Ccr2) expression level [40]. In addition, the chronic inflammation of large vessels subjected to rhythmic myeloid cell recruitment is regulated by the rhythmic release of myeloid cell-derived CCL2 [41]. All the available evidence suggests that chronopharmacology-based therapy targeting RORB, RORC, PER1, CRY1, or FTO might constitute another approach for the treatment of atherosclerosis.

Our analysis also identified four significantly changed types of immune cells (M0 macrophages, gamma delta T cells, plasma cells, and monocytes) in plaques with higher immune infiltration. According to the correlation analysis between genes and immune cells, seven genes (IGSF6, IL18, CECR1, FBP1, CAPG, HLA-DPA1 and MMP7) exhibited good correlation with these immune cells. Previous studies have demonstrated that the interruption of IL18 function reduces atherosclerosis in mice [42], and loss-of-function mutations in CECR1 could lead to systemic vasculopathy or vasculitis [43]. In addition, CAPG modulates the protective effects of unidirectional shear stress and might be related to the macrophage responses to oxidized LDL [44, 45]. However, fewer studies have focused on how these genes regulate immune cells in atherosclerosis. Further exploration of their functions in the pathogenesis of atherosclerosis might provide new methods for atherosclerosis therapy.

The innovation of this study is the exploration of different immune infiltration profiles, potential pathways and common genes associated with different immune cells in atherosclerosis using plaques from different vascular beds. Previous outstanding single-cell proteomic and transcriptomic study of plaques [46] and previous re-analysis of GSE100927 and GSE28829 [47] have only used the carotid plaques to identify the DEGs and to explore the immune infiltration, ignoring the heterogeneous nature of arteries from different peripheral vascular beds. The relationships between genes and different immune cells has not been explored in the previous study which has re-analysed GSE100927 and GSE28829 either [47]. Hence, our study might be more rational for identifying common cellular and molecular biological features in the course of atherosclerosis by pooling plaques from different vascular beds together. In addition, the classification method for atheromatous plaques based on immune infiltration calculated by ssGSEA and ESTIMATE algorithm in our study might be complementary to traditional clinical classification methods based on gross pathology and histopathology. The proposed classification method might provide us a better understanding of the molecular pathophysiological procedure of atherosclerosis.

However, our study has some limitations. First, there is no detail clinical information pertaining to the exact clinical stages of plaques in GSE100927. Hence, our hypothesis that plaques with higher immune infiltration might be at a more advanced stage of atherosclerosis, just like the results in GSE28829, needs to be cross validated in further study. Second, the results of this study are based on an analysis of gene expression values obtained from microarrays, and gene expression might not be directly equivalent to protein expression. Both in vitro and in vivo experiments should be performed to validate our findings at the gene transcription and translation levels. In addition, because the techniques of using in vivo or in vitro models for investigating the interaction between different immune cells and stromal cells (including vascular endothelial cells and smooth muscle cells) are immature, next-generation sequencing and proteomics studies of atheromatous plaques from different vascular beds with larger sample sizes might increase the confidence of our results.

## Conclusions

Taken together, the results obtained in our study provide novel insight into atherosclerosis: dysregulated in situ immune responses, loss of circadian rhythm, and abnormal m6A modification might orchestrate and lead to the progression of atherosclerosis. Chronopharmacology-based treatment targeting different immune cells (macrophage M0 and T cells gamma delta, plasma cells and monocytes) through different immune-related genes (CAPG, IGSF6, IL18, CECR1, FBP1, MMP7, HLA-DPA1, FTO, CRY1, RORB, and PER1) and immune-related pathways (IFN-γ pathway and PD-1 signalling pathway) might serve as potential therapies for atherosclerosis.

## Supplementary Information

**Additional file 1. Supplementary Figure 1: **Consensus clustering analysis of atherosclerosis samples in GSE100927. (A) Plot showing the results from the principal component analysis (PCA) of the gene expression profiles of 29 atheromatous plaques from the carotid arteries, 26 atheromatous plaques from the femoral arteries, 14 atheromatous plaques from the infrapopliteal arteries, 12 healthy control samples from the carotid arteries, 12 healthy control samples from the femoral arteries, and 11 healthy control samples from the infrapopliteal arteries in the GSE100927 dataset. (B) Cumulative distribution function (CDF) curve obtained from the consensus clustering analysis with k = 2–6 based on the Euclidean distance of the ssGSEA scores using the k-means clustering method. (C) Relative change in the area under the CDF curve obtained with k = 2–6. (D) Tracking plot for k = 2–6. (E)–(H) Consensus clustering matrices of k = 2, 3, 5, 6.

**Additional file 2. Supplementary Figure 2: **Identification of immune infiltration in different atheromatous plaques in GSE100927. (A) Boxplot comparing the relative proportion of 21 types of infiltrated immune cells between the ImmuneScoreL cluster and the ImmuneScoreH cluster. (B) Heatmap of the relative proportion of 21 types of infiltrated immune cells calculated using the CIBERSORT algorithm. (C) Correlations between 21 types of infiltrated immune cells. Pearson correlation coefficients are indicated in the upper triangular matrix. The p values are indicated in the lower triangular matrix: blank: p > 0.05, *: p <= 0.05, **: p <= 0.01, ***: p <= 0.001, ****: p <= 0.0001.

**Additional file 3. Supplementary Figure 3: **Atherosclerosis subtypes and distinct immune cell infiltration of samples in GSE28829. (A) Heatmap of ssGSEA scores of all 29 atheromatous carotid plaques in GSE28829 that were clustered based on the Euclidean distance using the Ward.D2 method. (B) Cluster dendrogram of ssGSEA scores of the 29 atheromatous carotid plaques based on the Euclidean distance using Ward.D2 method. (C) Cumulative distribution function (CDF) curve obtained from the consensus clustering analysis with k = 2–6 based on the Euclidean distance of the ssGSEA scores using the k-means clustering method. (D) Relative change in the area under the CDF curve obtained with k = 2–6. (E) Heatmap of the ssGSEA scores of plaques from the two subgroups in Figure S3B-C, as well as of the ImmuneScoreL cluster and the ImmuneScoreH cluster. (F)Boxplot comparing the relative proportion of 21 types of infiltrated immune cells calculated by CIBERSORT between the ImmuneScoreL cluster and the ImmuneScoreH cluster. (G) The comparison of immune infiltration in health controls to atheromatous plaques derived from GSE28829 and GSE100927 which were calculated after removing batch effects.

**Additional file 4. Supplementary Figure 4: **Gene set enrichment analysis (GSEA) comparing atheromatous plaques in the ImmuneScoreH cluster with those in the ImmuneScoreL cluster of GSE28829. (A-F) KEGG canonical pathways were used as the a priori knowledge for the GSEA. (A) Primary immunodeficiency, (B) leukocyte transendothelial migration, (C) antigen processing and presentation, (D) natural killer cell-mediated cytotoxicity, (E) T cell receptor signalling pathway, and (F) B cell receptor signalling pathway were highly enriched in the ImmuneScoreH cluster. (G-L) The REACTOME subset of canonical pathways was used as the a priori information for the GSEA. (G-J) Interferon-related pathways, (K) PD-1 signalling, and (L) neutrophil degranulation were highly enriched in the ImmuneScoreH cluster.

**Additional file 5. Supplementary Figure 5: **Comparison of the expression of genes related to different immune regulators in GSE28829. ns: p > 0.05, *: p <= 0.05, **: p <= 0.01, ***: p <= 0.001, ****: p <= 0.0001. Supplementary Figure 5A–E were a set of comparisons between atheromatous plaques in the ImmuneScoreH cluster with that in atheromatous plaques in the ImmuneScoreL cluster: box plot comparing the expression of human leukocyte antigen (HLA)-related genes(S5A), immune checkpoint-related genes (S5B), IFN-γ pathway marker genes(S5C), m6A methylation regulator-related genes(S5D), circadian rhythm-related genes(S5E). Figure S5F summarized the consistent results between the GSE100927 and GSE28829 (ImmunceScoreH cluster vs. ImmunceScoreL cluster).

**Additional file 6.** **Supplementary Table 1:** Gene of interest defined based on prior biological knowledge.

**Additional file 7.** **Supplementary Table 2:** DEGs between ImmuneScoreH cluster and other samples of GSE100927.

**Additional file 8.** **Supplementary Table 3:** DEGs between ImmuneScoreH cluster and ImmuneScoreL cluster of GSE100927.

## Data Availability

The dataset GSE100927 and dataset GSE28829 analysed during the current study are available in the Gene Expression Omnibus repository. Persistent web links to these datasets are as follow: GSE100927: https://www.ncbi.nlm.nih.gov/geo/query/acc.cgi?acc=GSE100927; GSE28829: https://www.ncbi.nlm.nih.gov/geo/query/acc.cgi?acc=GSE28829.

## Abbreviations

AS: Atherosclerosis
m6A: N6-methyladenosine
GEO: Gene expression omnibus
ssGSEA: Single-sample gene-set enrichment analysis
FC: Fold-changes
DEGs: Differentially expressed genes
GO: Gene ontology
KEGG: Kyoto encyclopedia of genes and genomes
BP: Biological process
CC: Cellular components
PPI networks: Protein–protein interaction networks
GSEA: Gene set enrichment analysis
NES: Normalized enrichment score
FDR: False discovery rate
PCA: Principle component analysis
CDF: Cumulative distribution function
ImmuneScoreH: High immune score
ImmuneScoreL: Low immune score
HLA: Human leukocyte antigen
CD: Clusters of differentiation
IFN-γ: Interferon gamma
CTLA4: Cytotoxic T-lymphocyte-associated protein 4(also known as CD152)
HAVCR2: Hepatitis A virus cellular receptor 2 (also known as T-cell immunoglobulin and mucin-domain containing-3)
ICOS: Inducible T-Cell co-stimulator
IDO1: Indoleamine 2,3-dioxygenase 1
LAG3: Lymphocyte cctivation gene 3
PDCD1: Programmed cell death-1 (also known as PD-1, CD279)
PDCD1LG2: Programmed cell death-1 ligand-2 (also known as PD-L2, CD273)
PDCD1LG1: Programmed cell death-1 Ligand-1(also known as PD-L1, CD274)
TIGIT: T cell immunoreceptor with Ig And ITIM domains
YTHDC1: YTH domain containing protein 1
METTL3/14: Methyltransferase-like protein 3/14
FTO: Alpha-ketoglutarate-dependent dioxygenase FTO (also known as Fat mass and obesity-associated protein)
ZC3H13: Zinc finger CCCH domain-containing protein 13
HNRNPC: Heterogeneous nuclear ribonucleoprotein C
WTAP: WT1 associated protein
ALKBH5: AlkB Homolog 5
YTHDF1/2: YTH N6-methyladenosine RNA binding protein 1/2
NR1D1/2: Nuclear receptor subfamily 1 Group D Member 1/2
SIRT1: Sirtuin 1
FBXL3: F-box/LRR-repeat protein 3
RORA: RAR related orphan receptor A
RORB: RAR related orphan receptor B
RORC: RAR related orphan receptor C
CRY1/2: Cryptochrome circadian regulator 1/2
CLOCK: Circadian locomoter output cycles kaput protein
ARNTL: Aryl hydrocarbon receptor nuclear translocator-like protein 1(also known as BMAL1)
PER1/2/3: Period circadian regulator 1/2/3
SUMO3: Small ubiquitin like modifier 3
CSNK1A1: Casein kinase-1 alpha-1
MAPK: Mitogen-activated protein kinase
ERK: Extracellular signal-regulated kinase
JNK: JUN N-terminal kinase
E4BP4: E4 promoter-binding protein 4 (also known as NFIL3)
DBP: Albumin D-Box binding protein
CSNK1E: Casein kinase 1 epsilon
CSNK1D: Casein kinase 1 delta
GSK3B: Glycogen synthase kinase 3 beta
BTRC: Beta-transducin repeat containing E3 ubiquitin protein ligase
IFNGR1/2: Interferon gamma receptor 1/2
IFNG: Interferon gamma
STAT1: Signal transducer and activator of transcription-1
JAK1/2: Janus kinase1/2
PTPN6: Protein tyrosine phosphatase non-receptor type-6
VAMP8: Vesicle associated membrane protein 8
ITGB2: Integrin subunit beta 2
ITGAM: Integrin subunit alpha-M
CXCL1: C-X-C motif chemokine ligand-1
CYBA: Cytochrome B-245 alpha chain
CYBB: Cytochrome B-245 beta chain (also known as NADPH Oxidase 2)
FCER1G: Fc receptor gamma-chain
LCK: Lymphocyte cell-specific protein-tyrosine kinase
PTPRJ: Protein tyrosine phosphatase receptor type-J (also known as SCC1 or DEP1)
CAPG: Actin regulatory protein CAP-G
IGSF6: Immunoglobulin superfamily member-6
IL18: Interleukin-18
CECR1: Cat eye syndrome critical region protein-1
FBP1: Fructose-bisphosphatase-1
MMP7: Matrix metallopeptidase-7
