# Supplementary figures and images for: Identification of potential therapeutic targets for atherosclerosis by analysing the gene signature related to different immune cells and immune regulators in atheromatous plaques
Source: BMC Med Genomics. 2021 Jun 3;14:145. doi: 10.1186/s12920-021-00991-2 (PMC8176741; doi:10.1186/s12920-021-00991-2)

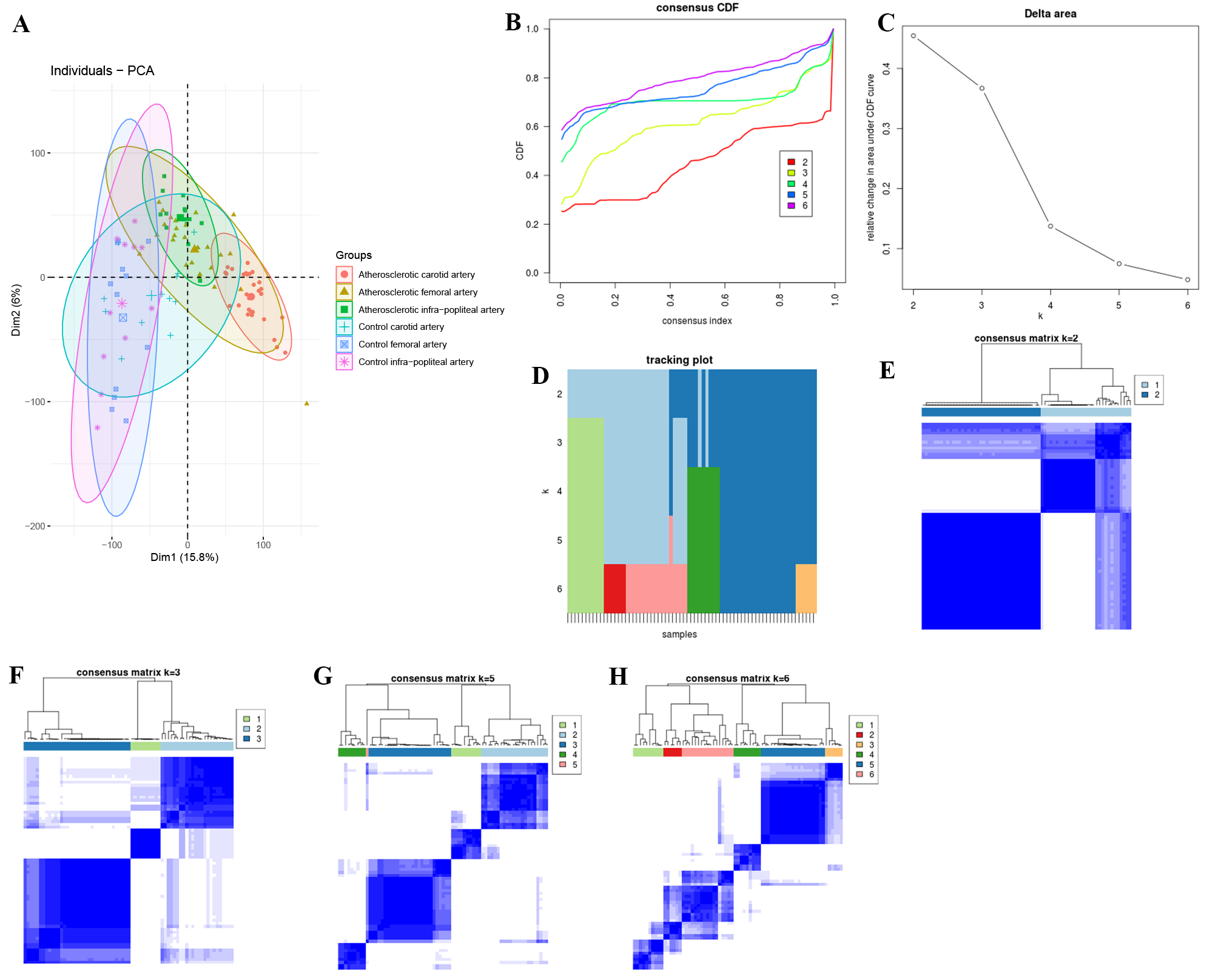

Supplement: Supplementary file 1 — Additional file 1. Supplementary Figure 1: Consensus clustering analysis of atherosclerosis samples in GSE100927. (A) Plot showing the results from the principal component analysis (PCA) of the gene expression profiles of 29 atheromatous plaques from the carotid arteries, 26 atheromatous plaques from the femoral arteries, 14 atheromatous plaques from the infrapopliteal arteries, 12 healthy control samples from the carotid arteries, 12 healthy control samples from the femoral arteries, and 11 healthy control samples from the infrapopliteal arteries in the GSE100927 dataset. (B) Cumulative distribution function (CDF) curve obtained from the consensus clustering analysis with k = 2–6 based on the Euclidean distance of the ssGSEA scores using the k-means clustering method. (C) Relative change in the area under the CDF curve obtained with k = 2–6. (D) Tracking plot for k = 2–6. (E)–(H) Consensus clustering matrices of k = 2, 3, 5, 6. [file 12920_2021_991_MOESM1_ESM.tif]

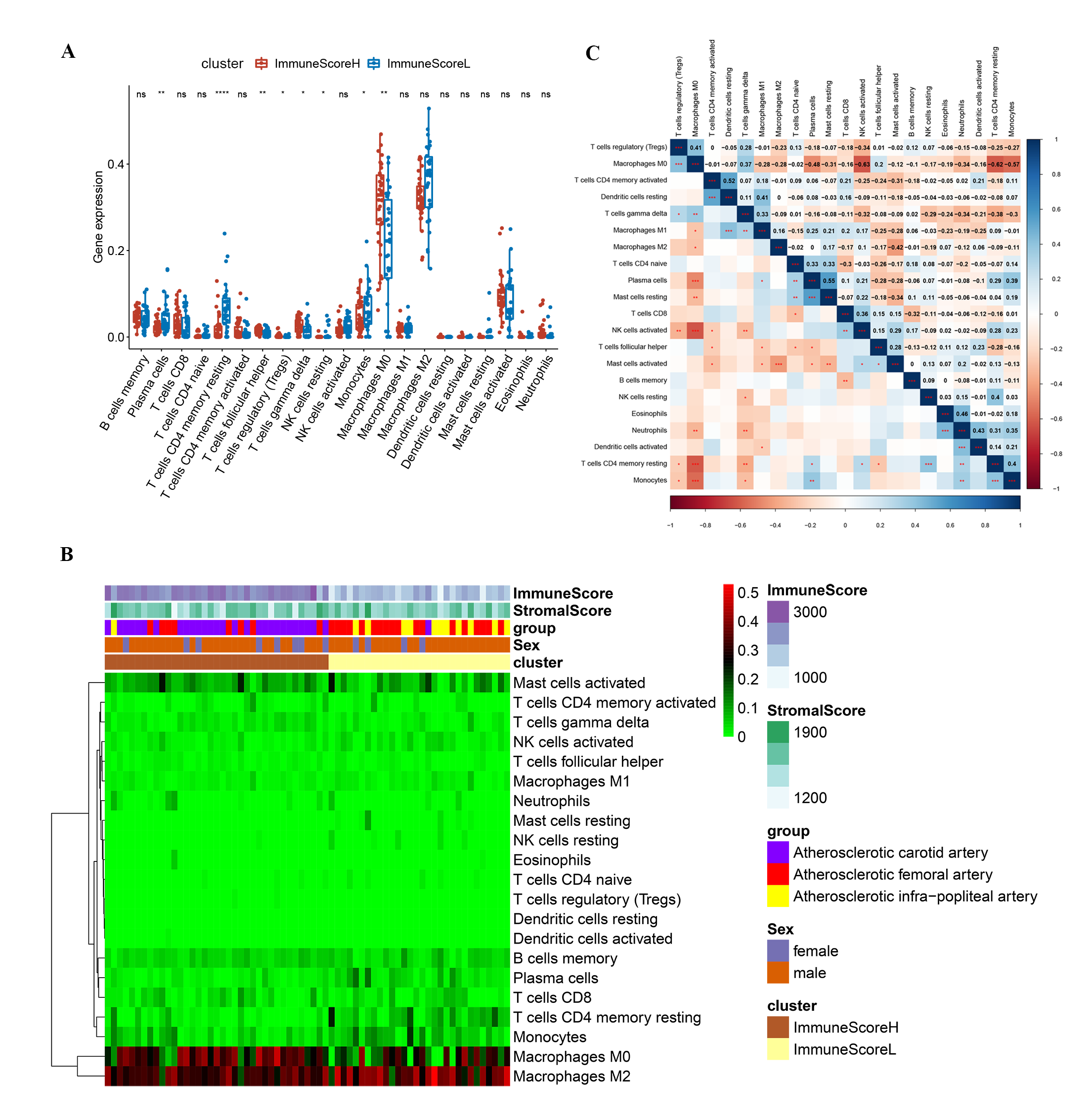

Supplement: Supplementary file 2 — Additional file 2. Supplementary Figure 2: Identification of immune infiltration in different atheromatous plaques in GSE100927. (A) Boxplot comparing the relative proportion of 21 types of infiltrated immune cells between the ImmuneScoreL cluster and the ImmuneScoreH cluster. (B) Heatmap of the relative proportion of 21 types of infiltrated immune cells calculated using the CIBERSORT algorithm. (C) Correlations between 21 types of infiltrated immune cells. Pearson correlation coefficients are indicated in the upper triangular matrix. The p values are indicated in the lower triangular matrix: blank: p > 0.05, *: p <= 0.05, **: p <= 0.01, ***: p <= 0.001, ****: p <= 0.0001. [file 12920_2021_991_MOESM2_ESM.tif]

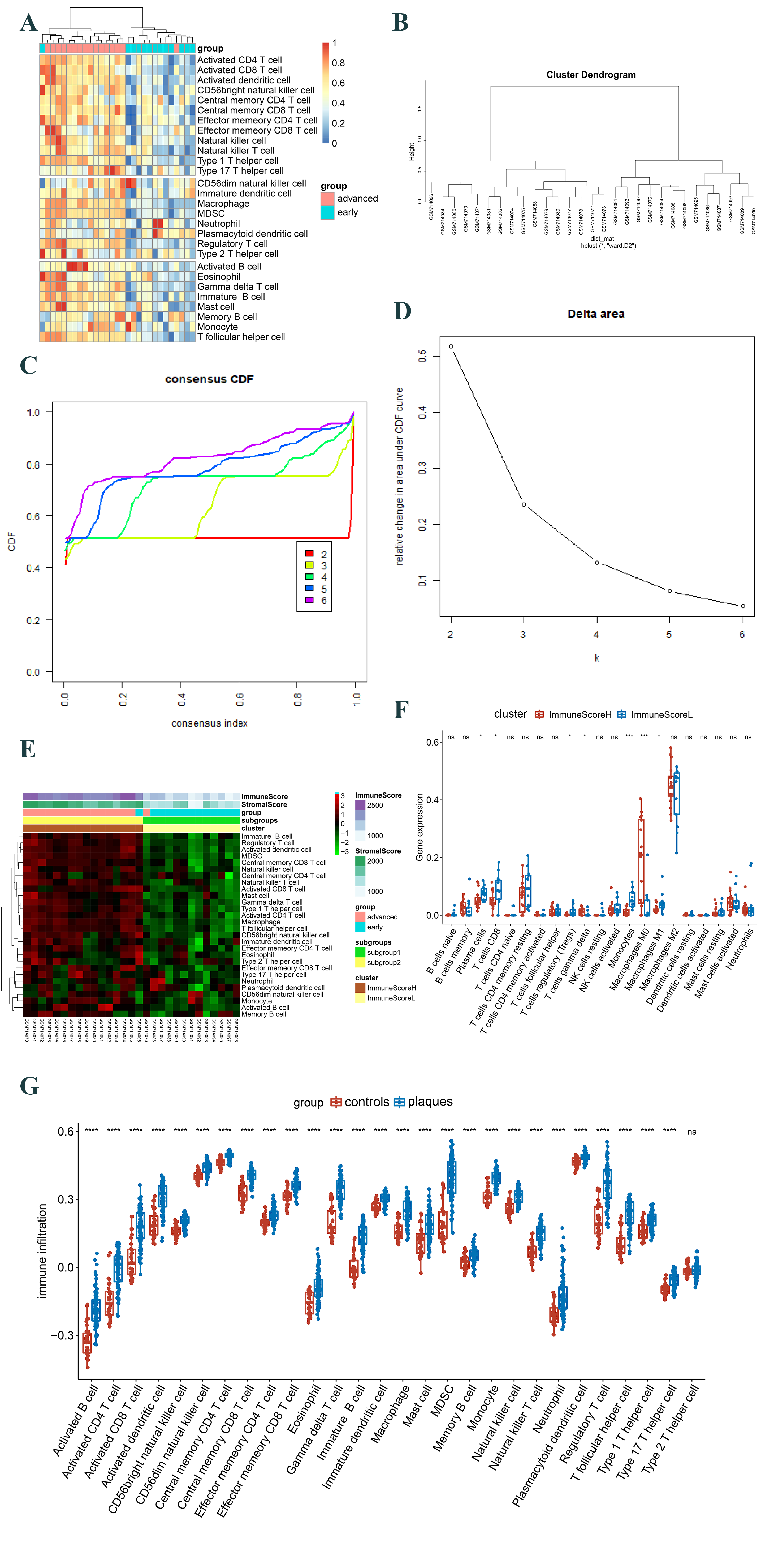

Supplement: Supplementary file 3 — Additional file 3. Supplementary Figure 3: Atherosclerosis subtypes and distinct immune cell infiltration of samples in GSE28829. (A) Heatmap of ssGSEA scores of all 29 atheromatous carotid plaques in GSE28829 that were clustered based on the Euclidean distance using the Ward.D2 method. (B) Cluster dendrogram of ssGSEA scores of the 29 atheromatous carotid plaques based on the Euclidean distance using Ward.D2 method. (C) Cumulative distribution function (CDF) curve obtained from the consensus clustering analysis with k = 2–6 based on the Euclidean distance of the ssGSEA scores using the k-means clustering method. (D) Relative change in the area under the CDF curve obtained with k = 2–6. (E) Heatmap of the ssGSEA scores of plaques from the two subgroups in Figure S3B-C, as well as of the ImmuneScoreL cluster and the ImmuneScoreH cluster. (F)Boxplot comparing the relative proportion of 21 types of infiltrated immune cells calculated by CIBERSORT between the ImmuneScoreL cluster and the ImmuneScoreH cluster. (G) The comparison of immune infiltration in health controls to atheromatous plaques derived from GSE28829 and GSE100927 which were calculated after removing batch effects. [file 12920_2021_991_MOESM3_ESM.tif]

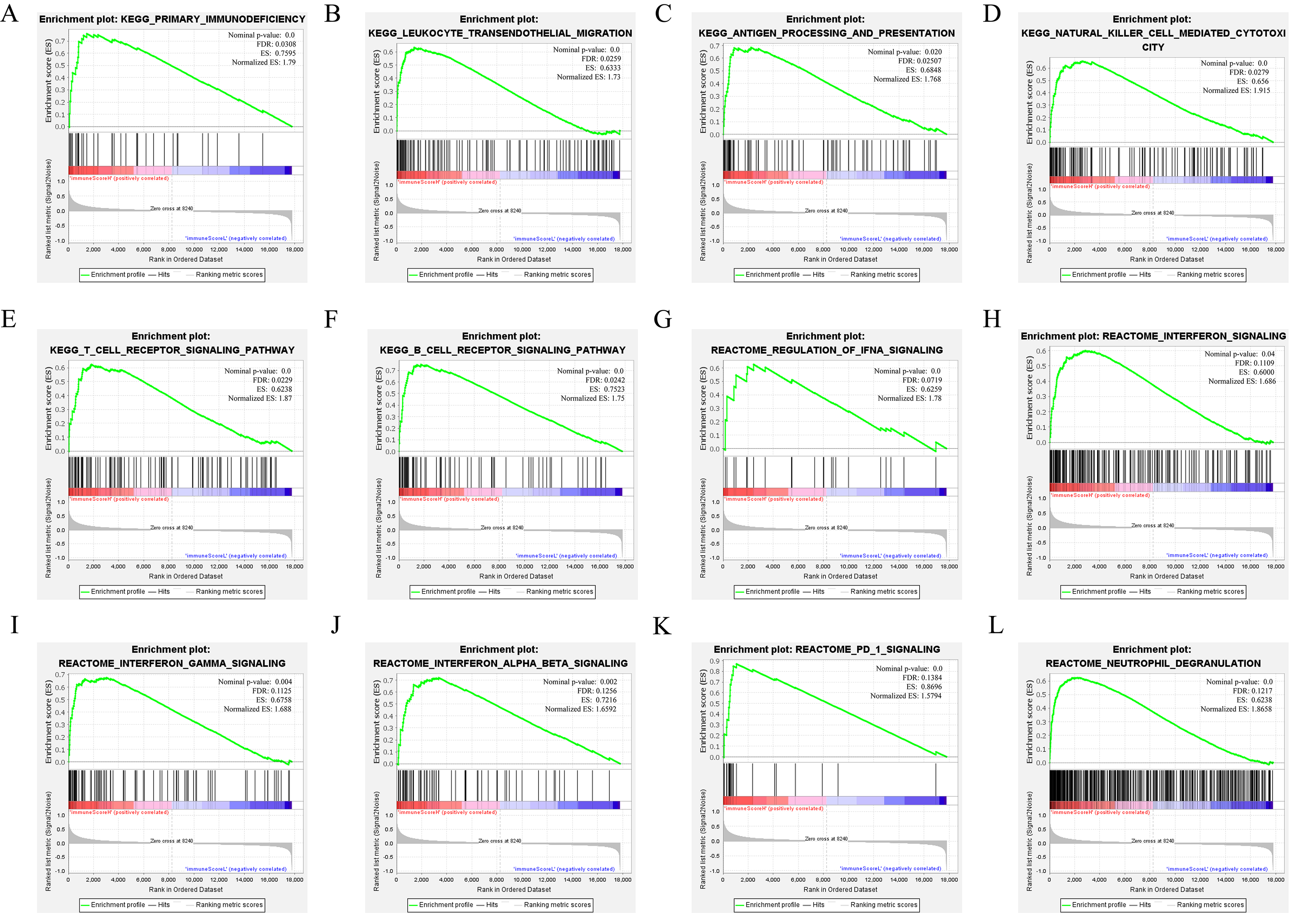

Supplement: Supplementary file 4 — Additional file 4. Supplementary Figure 4: Gene set enrichment analysis (GSEA) comparing atheromatous plaques in the ImmuneScoreH cluster with those in the ImmuneScoreL cluster of GSE28829. (A-F) KEGG canonical pathways were used as the a priori knowledge for the GSEA. (A) Primary immunodeficiency, (B) leukocyte transendothelial migration, (C) antigen processing and presentation, (D) natural killer cell-mediated cytotoxicity, (E) T cell receptor signalling pathway, and (F) B cell receptor signalling pathway were highly enriched in the ImmuneScoreH cluster. (G-L) The REACTOME subset of canonical pathways was used as the a priori information for the GSEA. (G-J) Interferon-related pathways, (K) PD-1 signalling, and (L) neutrophil degranulation were highly enriched in the ImmuneScoreH cluster. [file 12920_2021_991_MOESM4_ESM.tif]

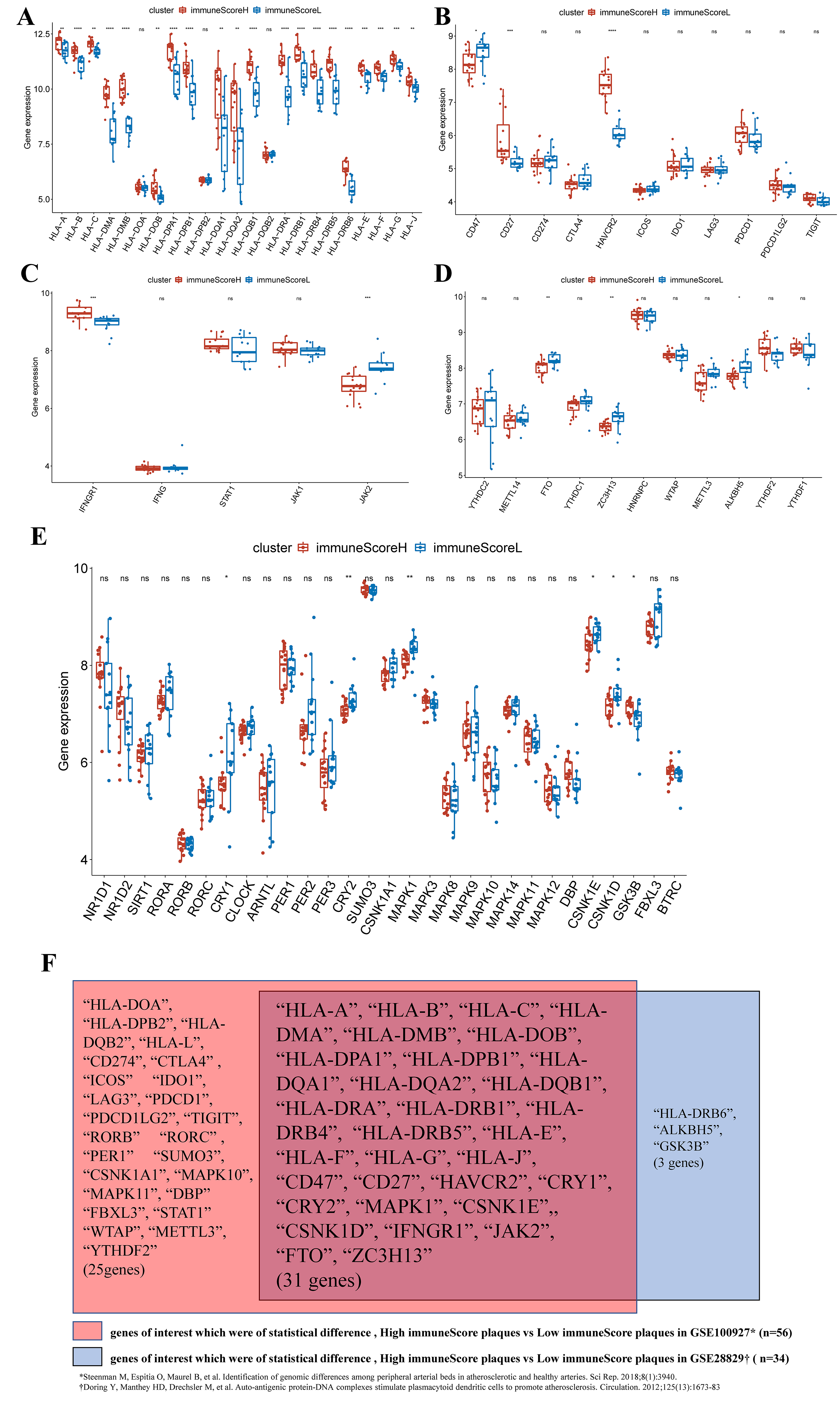

Supplement: Supplementary file 5 — Additional file 5. Supplementary Figure 5: Comparison of the expression of genes related to different immune regulators in GSE28829. ns: p > 0.05, *: p <= 0.05, **: p <= 0.01, ***: p <= 0.001, ****: p <= 0.0001. Supplementary Figure 5A–E were a set of comparisons between atheromatous plaques in the ImmuneScoreH cluster with that in atheromatous plaques in the ImmuneScoreL cluster: box plot comparing the expression of human leukocyte antigen (HLA)-related genes(S5A), immune checkpoint-related genes (S5B), IFN-γ pathway marker genes(S5C), m6A methylation regulator-related genes(S5D), circadian rhythm-related genes(S5E). Figure S5F summarized the consistent results between the GSE100927 and GSE28829 (ImmunceScoreH cluster vs. ImmunceScoreL cluster). [file 12920_2021_991_MOESM5_ESM.tif]
