# Supplementary material for: Identification of potential therapeutic targets for atherosclerosis by analysing the gene signature related to different immune cells and immune regulators in atheromatous plaques
Source: BMC Med Genomics. 2021 Jun 3;14:145. doi: 10.1186/s12920-021-00991-2 (PMC8176741; doi:10.1186/s12920-021-00991-2)
Supplement: Supplementary file 6 — Additional file 6. Supplementary Table 1: Gene of interest defined based on prior biological knowledge. [file 12920_2021_991_MOESM6_ESM.docx]

**Supplementary table 1. Genes of interest**

| **Categories** | **Gene Symbols** |
| --- | --- |
| HLA molecules related genes | 'HLA-A', 'HLA-B', 'HLA-C', 'HLA-DMA', 'HLA-DMB', 'HLA-DOA', 'HLA-DOB', 'HLA-DPA1', 'HLA-DPA2', 'HLA-DPA3', 'HLA-DPB1', 'HLA-DPB2', 'HLA-DQA1', 'HLA-DQA2', 'HLA-DQB1', 'HLA-DQB2', 'HLA-DQB3', 'HLA-DRA', 'HLA-DRB1', 'HLA-DRB2', 'HLA-DRB3', 'HLA-DRB4', 'HLA-DRB5', 'HLA-DRB6', 'HLA-DRB7', 'HLA-DRB8', 'HLA-DRB9', 'HLA-E', 'HLA-F', 'HLA-G', 'HLA-H', 'HLA-J', 'HLA-K', 'HLA-L', 'HLA-N', 'HLA-P', 'HLA-S', 'HLA-T', 'HLA-U', 'HLA-V', 'HLA-W', 'HLA-X', 'HLA-Y', 'HLA-Z' |
| Immune checkpoint molecules related genes | 'CD47','CD27', 'CD274', ‘PD-L1，'PDCD1LG1', 'CTLA4', 'HAVCR2', 'ICOS', 'IDO1', 'LAG3', 'PDCD1', 'PDCD1LG2', 'TIGIT' |
| m6A regulators related genes | 'YTHDC2', 'METTL14', 'FTO', 'YTHDC1', 'ZC3H13', 'HNRNPC', 'WTAP', 'METTL3', 'ALKBH5', 'YTHDF2', 'YTHDF1' |
| Circadian rhythm related genes | 'NR1D1', 'NR1D2', 'SIRT1', 'RORA', 'RORB', 'RORC', 'CRY1', 'CLOCK', 'ARNTL', 'BMAL1', 'PER1', 'PER2', 'PER3', 'CRY2', 'SUMO3', 'CSNK1A1', 'MAPK1', 'MAPK3', 'ERK1', 'ERK2', 'MAPK8’, 'JNK1', 'MAPK9', 'JNK2', 'MAPK10', 'JNK3', 'MAPK14', 'MAPK11', 'MAPK12', 'ERK6', 'E4BP4', 'DBP', 'CSNK1E', 'CSNK1D', 'GSK3B', 'FBXL3', 'BTRC' |
| IFN-γ signaling pathway related genes | 'IFNGR2', 'IFNGR1', 'IFNG', 'STAT1', 'JAK1', 'JAK2' |

The genes of interest are defined based on prior biological knowledge, e.g., published information about biochemical pathways or coexpression in previous experiments.
